# Supplementary material for: Spirulina maxima Derived Pectin Nanoparticles Enhance the Immunomodulation, Stress Tolerance, and Wound Healing in Zebrafish
Source: Mar Drugs. 2020 Nov 7;18(11):556. doi: 10.3390/md18110556 (PMC7695216; doi:10.3390/md18110556)
Supplement: Supplementary file 1 [file marinedrugs-18-00556-s001.pdf]

Supplementary table 1. Gene specific primers used in this study.

Gene name	Accession number	Primer name	Primer sequence (5'-3')
**Zebrafish genes**			
Toll like receptor 2 (*tlr2*)	NM_212812.1	tlr2-F	TCTCCGTCTTGGTTTCAC
		tlr2-R	GGTCCCACAGTTGAGTATG
Toll like receptor 4b (*tlr4b*)	AY388400.1	tlr4b-F	GGAATAATGGGCAGCCGTAAG
		tlr4b-R	AGCGACACCAACTATCAATG
Toll like receptor 5 (*tlr5b*)	BC163185.1	tlr5b-F	GAAACATTCACCCTGGCACA
		tlr5b-R	CTACAACCAGCACCACCAGAATG
crel	AY163837	crel-F	ACTACAGCTCCCAACAGCCTCAAA
		crel-R	AAACTGGTAGCCCGTTGCTAGTGA
Interleukin-1β (*il1β*)	AY340959.1	il1β-F	TCAAACCCCAATCCACAGAG
		il1β-R	TCACTTCACGCTCTTGGATG
Tumor necrosis factor- α (*tnfα*)	AY427649	tnfα-F	AGAAGGAGAGTTGCCTTTACCGCT
		tnfα-R	AACACCCTCCATACACCCGACTTT
Interleukin-6 (*il6*)	JN698962.1	il6-F	TCAACTTCTCCAGCGTGATG
		il6-R	TCTTTCCCTCTTTTCCTCCTG
Interleukin-10 (*il10*)	AY887900.1	il10-F	CCCTATGGATGTCACGTCATG
		il10-R	CATATCCCGCTTGAGTTCCTG
Chemokine ligand 8a (*cxcl8a*)	XM_009306855.2	cxcl8a-R	CTTCCCTCCAAGCCCACAC
		cxcl8a-R	GATCCGGGCATTCATGG
Chemokine ligand 18b (*cxcl18b*)	NM_001115060	cxcl18b-F	CTGCTGCTGCCGGTAGTTTA
		cxcl18b-R	TCAACTTTGTCGCAGTTTGG
CC-chemokine (*ccl34a.4*)	BC162421.1	ccl34 a.4-F	TGCAGCTCAACCAGAAGATG
		ccl34 a.4-R	CTTTGACGCATGGAGGATTT
Chemokines CCL-C24 (*ccl34b.4*)	NP_001108521	ccl34b.4-F	TCGAGTTGGAGTAACATGTG
		ccl34b.4-R	GTACCTTTCCTTCTCTGCGTAG
Defensin, beta-like1 (*defβl1*)	NM_001081553.1	defbl1-F	CTGCTTGTCCTTGTCGTACT
		defbl1-R	GCAAACACACTCCTTGTCTG
Hepcidin (*hamp*)	NM_205583.2	hamp-F	CATACAGCAGGTACAGGATGAG
		hamp-R	GAGGGTCTGTTAGTCTGTGTTC
Cathepsin D (*ctsd*)	NM_ 131710.2	ctsd-F	GAACCCTCAGACGAACACTAAG
		ctsd-R	GGGAAGCCCAGGTTGTATTT
Lysozyme-C (*lyz*)	AF402599	lyz-F	AAGCAGGTTTAAGACCCACCGAGT
		lyz-R	AAGTCTGAACAGGCCACTTTGCAC
Alkaline phosphatase (*alp*)	JX847415.1	alp-F	GGTCGTACAACTGCTTATCCTC
		alp-R	GAGCAAAGTCCCTCCAGTAATC
Mucin 2.1 (*muc2.1*)	NC_007136.7	muc 2.1-F	AATATGCCTTGCGGAACAAC
		muc 2.1-R	GTGCTGAGGTTGCAGAATGA
Mucin 5.1 (*muc5.1*)	XM_009297795.1	muc 5.1-F	TGGCAACTTGGCTGATGATA
		muc 5.1-R	TCGTCACACGGACCAGTAGA
Mucin 5.2 (*muc5.2*)	XM_009297793.1	muc 5.2-F	GGTGTCTGTTCCGATCAATC
		muc 5.2-R	TCATCCTTGTCGCCATTGTA
Mucin 5.3 (*muc5.3*)	ENSDARG00000089847	muc 5.3-F	GGGGAAAACTACACCAGCAA
		muc 5.3-R	TGTGAATTCTGTGCCAGAGC
Heat shock protein (*hsp70*)	AB062116.1	hsp70-F	CATGGTCCTGGTGAAGATGAA
		hsp70-R	GTCTGTGGGACTCGTTGAAATA
Heat shock protein (*hsp90ab1*)	NM_131310.3	hsp90ab1-F	GAAGAGGAGAAGGCAGAGAAAG
		hsp90ab1-R	CGAGCCGACATCTTCAATCT
Transforming growth factor, β1 (*tgfβ1*)	XM_687246	tgfβ1-F	CCCAAGGAACCAGAAGTAGAAG
		tgfβ1-R	GGATCTTCTATGGTGTGCTGAA
Matrix metalloproteinase 9 (*mmp9*)	AY151254	mmp9-F	TTTGCCCTGATCGTGGATAC
		mmp9-R	GGGAAACCCTCCACGTATTT
Matrix metalloproteinase 13 (*mmp13*)	AF506756	mmp13-F	GAGAAGGTTTGGGCTCTCTATG
		mmp13-R	TGAGTTGCTGTCTTCCTTGTAG
Tissue inhibitor of metalloproteinase 2 (*timp2b*)	BC056534	timp2b-F	GCGTTATGTGGAGTCAGTCTT
		timp2b-R	CTCGATGTAGTCACACAGGTTTAT
Super oxide dismutase (*sod1*)	NM_131294.1	sod1-F	AGGTGACTGGTGAAATTACTGG
		sod1-R	GTCTCACACTATCGGTTGGC
Catalase (*cat*)	NM_130912.2	cat-F	CCAAGGTCTGGTCCCATAAAG
		cat-R	GCTCAACCTCCGCGAAATA
β- actin	AF025305	β actin- F	AATCTTGCGGTATCCACGAGACCA
		β actin- R	TCTCCTTCTGCATCCTGTCAGCAA

Mouse genes			
Toll-like receptor 4 (Tlr4)	NM_021297.3	Tlr4-F	GCTTACACCACCTCTCAAACT
		Tlr4-R	ACAGCCACCAGATTCTCTAAAC
Myeloid differentiation primary response gene 88 (Myd88)	NM_010851.3	Myd88-F	TCGATGCCTTTATCTGCTACTG
		Myd88-R	GGTCGGACACACACAACTTA
Interleukin 6, transcript variant 1 (Il 6)	NM_031168.2	Il6-F	CTTCCATCCAGTTGCCTTCT
		Il6-R	CTCCGACTTGTGAAGTGGTATAG
Interleukin 10 (Il 10)	NM_010548.2	Il10-F	TTGAATTCCCTGGGTGAGAAG
		Il10-R	TCCACTGCCTTGCTCTTATTT
Chemokine (C-X-C motif) ligand transcript variant 2 (Cxcl 12)	NM_013655.4	Cxcl12-F	CTCTGCATCAGTGACGGTAAA
		Cxcl12-R	CACAGTTTGGAGTGTTGAGGA
Chemokine (C-C motif) ligand 3 (Ccl 3)	NM_011337.2	Ccl3-F	GAAGATTCCACGCCAATTCATC
		Ccl3-R	GATCTGCCGGTTTCTCTTAGTC
Defensin, alpha, 21 (Def 1)	NM_183253.3	Def1-F	CCAGGGGAAGATGACCAGGCTG
		Def1-R	TGCAGCGACGATTTCTACAAAGGC
Defensin, alpha, related sequence 2 (Def 2)	NM_007847.1	Def 2-F	GCATGGAATCTGGGTCAAGATAAC
		Def 2-R	AGAAGGAAGAGCAATCAAGGCTAAG
Lysozyme 1 (Lyz 1)	NM_013590.4	Lyz1-F	GAAGCACCGACTATGGGATATT
		Lyz1-R	GATCCCACAGGCATTCTTAGAT
Mucin 1 (Muc 1)	NM_013605.2	Muc1-F	GGCATTCGGGCTCCTTTCTT
		Muc1-R	TGGAGTGGTAGTCGATGCTAAG
Catalase (Cat)	NM_009804.2	Cat-F	GATGGTAACTGGGATCTTGTGG
		Cat-R	GTGGGTTTCTCTTCTGGCTATG
Super oxide dismutase (Sod 1)	NM_131294.1	Sod1-F	AGGTGACTGGTGAAATTACTGG
		Sod1-R	GTCTCACACTATCGGTTGGC
Superoxide dismutase 2; mitochondrial (Sod 2)	NM_013671.3	Sod2-F	AGCGTGACTTTGGGTCTTT
		Sod2-R	AGCGACCTTGCTCCTTATTG
Glyceraldehyde-3-phosphate dehydrogenase (Gapdh)	NM_001289726.1	Gapdh-F	AGGTCATCCCAGAGCTGAACG
		Gapdh-R	CACCCTGTTGCTGTAGCCGTAT

Supplementary figures

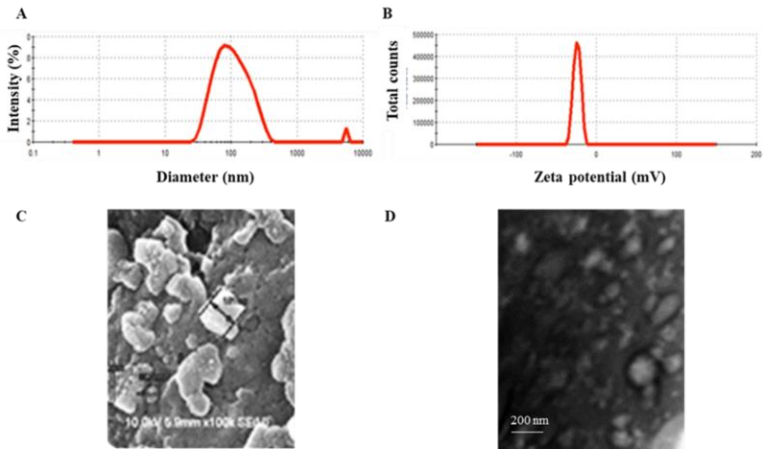

Sup. Fig. 1. Physicochemical properties of SmPNPs. A: size distribution, B: zeta potential. Morphology of SmPNPs under C: field-emission scanning electron microscopy (FE-SEM) and D: field-emission transmission electron microscopy (FE-TEM). FE-SEM and FE-TEM images show the irregular shape of SmPNPs.

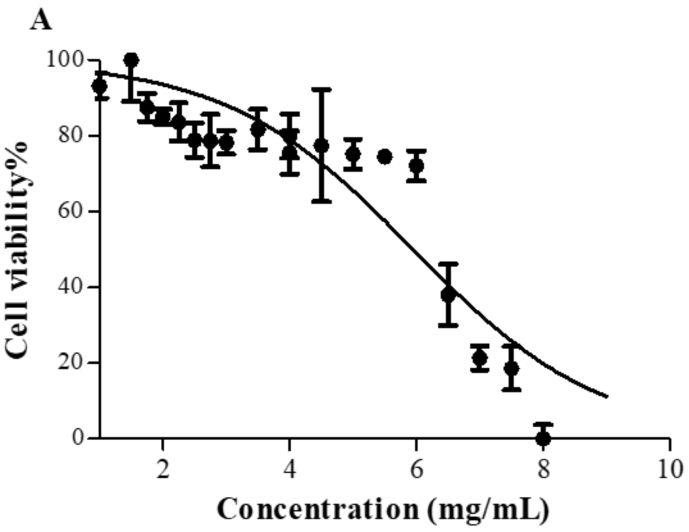

Sup. Fig. 2. *In vitro* cytotoxicity assay of SmPNPs on Raw 264.7. SmPNPs were treated with different concentrations (1-8 mg/mL) of SmPNPs, and cell viability% was evaluated. Values were presented as means plus or minus standard error (± SE) of three independent experiments, and each carried out in triplicates.

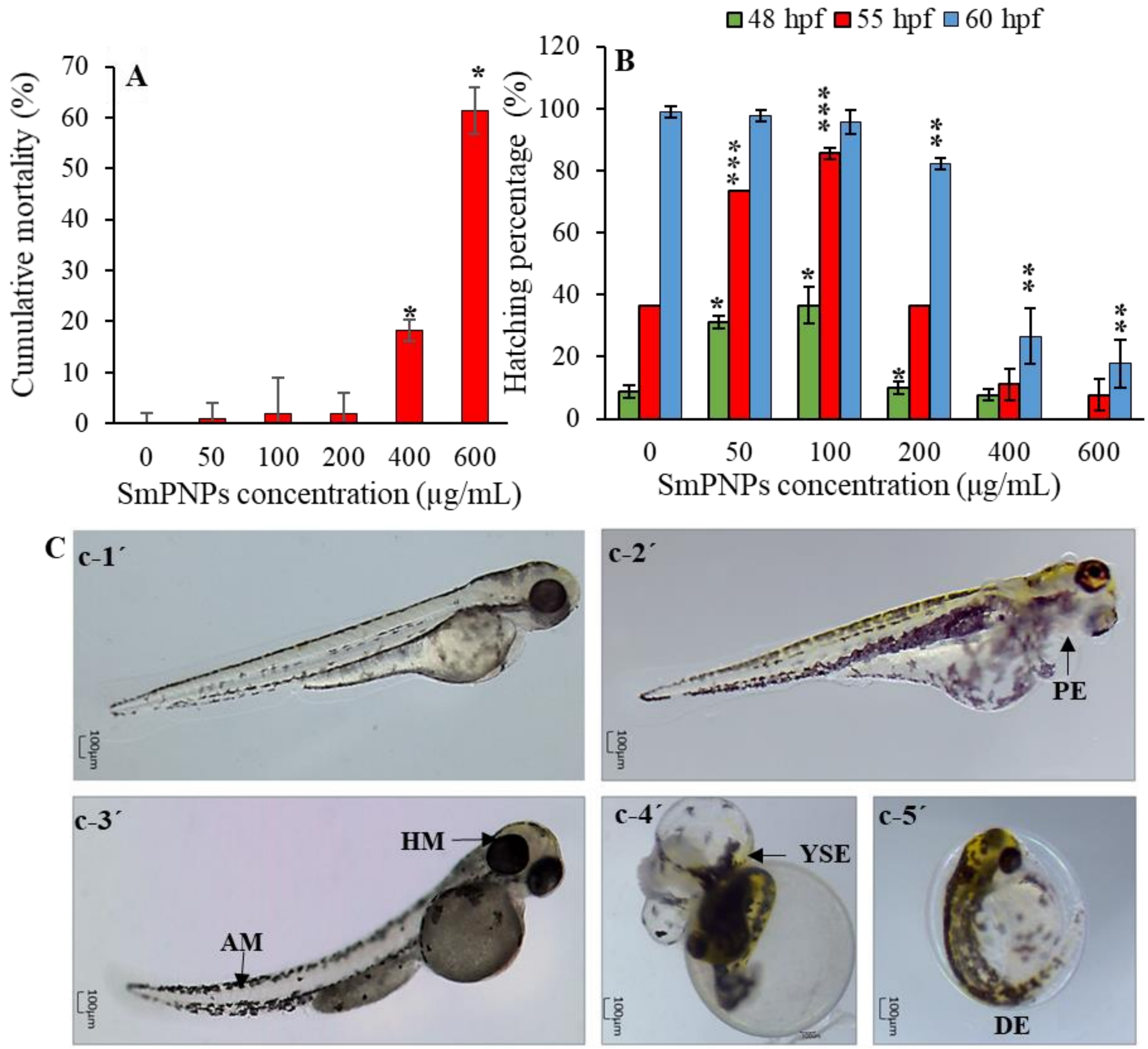

Sup. Fig. 3. *In vivo* toxicity of SmPNPs exposed to zebrafish embryos. A: cumulative mortality, B: hatching rate, C: malformations of embryos induced by SmPNPs, c-1´: control larva; c-2´: pericardial oedema (PE); c-3´: axial malformation (AE) and head malformation (HM); c-4´: yolk sack oedema (YSE); c-5´: dead embryo (DE). Data are expressed as means plus or minus standard error (± SE) from three independent experiments. Significant differences between experimental groups are denoted by asterisk (*) marks. (one-way and/or two-way ANOVA *P <.05, **P <.01, ***P <.001).

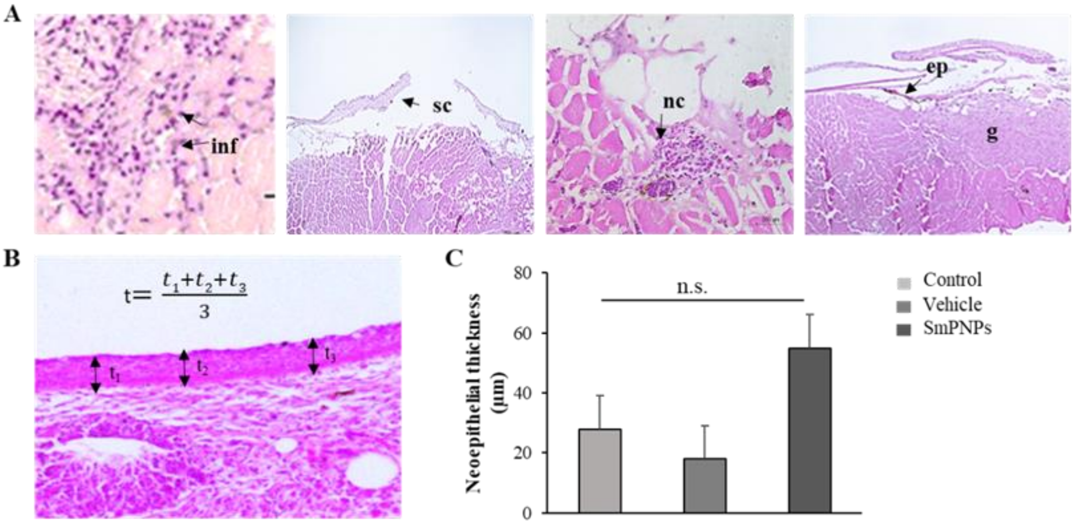

D

dpw	Group	Abundance of inflammatory cells (inf)	Scab (sc)	Necrosis and slough (nc)	Re-epithelialization (ep)	Formation of granulation tissue (g)
2	Control	+++	++	++	-	-
	Vehicle	+++	+++	++	-	-
	SmPNPs	++	++	+++	-	+
7	Control	+++	++	+	+	++
	Vehicle	++	++	+	+	+
	SmPNPs	++	+	-	++	+++

Sup. Fig. 4. Quantitative analysis for major histological parameters in SmPNPs wound healing. A: representative images used for semi-quantitative analysis, B: quantitative analyzed criteria for neoepithelial thickness in healing wounds. C: graphs showing the nonepithelial thickness. The values are the mean ± standard deviation (SD) (n = 3). Unpaired two-tailed t-test was performed to find statistical significance (*$P < .05$). D: Selected histological indexes demonstrating the diminishing of abundance of inflammatory cells (inf), scab (sc), necrosis and slough (nc), re-epithelialization (ep) and formation of granulation tissue (g) on wound surface at 2 and 7 dpw. (Notes: +, slight; ++, moderate; +++, marked; -, absence).
